# Investigation of the molecular genetic causes of non-syndromic primary ovarian ınsufficiency by next generation sequencing analysis

**DOI:** 10.20945/2359-4292-2022-0475

**Published:** 2023-12-01

**Authors:** Eren Er, Semih Aşıkovalı, Hatice Özışık, Elif Sağsak, Damla Gökşen, Hüseyin Onay, Füsun Saygılı, Şükran Darcan, Samim Özen

**Affiliations:** 1 Tepecik Training and Research Hospital Department of Pediatric Endocrinology Izmir Turkey Tepecik Training and Research Hospital, Department of Pediatric Endocrinology, Izmir, Turkey; 2 Ege University Faculty of Medicine Department of Medical Genetics Izmir Turkey Ege University Faculty of Medicine, Department of Medical Genetics, Izmir, Turkey; 3 Ege University Faculty of Medicine Department of Endocrinology and Metabolism Izmir Turkey Ege University Faculty of Medicine, Department of Endocrinology and Metabolism, Izmir, Turkey; 4 University of Health Sciences Gaziosmanpaşa Training and Research Hospital Clinic of Pediatric Endocrinology Istanbul Turkey University of Health Sciences, Gaziosmanpaşa Training and Research Hospital, Clinic of Pediatric Endocrinology, Istanbul, Turkey

**Keywords:** Primary ovarian insufficiency, candidate genes, next generation sequencing analysis

## Abstract

**Objective:**

The aim of this study is to investigate the molecular genetic causes of non-syndromic primary ovarian insufficiency (POI) cases with the gene panel based on next generation sequencing analysis and to establish the relationship between genotype and phenotype.

**Subjects and methods:**

Twenty three cases aged 14-40 years followed up with POI were included. Patients with a karyotype of 46, XX, primary or secondary amenorrhea before the age of 40, with elevated FSH (>40 IU/mL) and low AMH levels (<0.03 ng/mL) were included in the study. Molecular genetic analyzes were performed by the next generation sequencing analysis method targeted with the TruSight^TM^ Exome panel.

**Results:**

Median age of the cases was 17.8 (14.0-24.3) years, and 12 (52%) cases admitted before the age of 18. Fifteen (65%) patients had consanguineous parents. In 2 (8.6%) cases, variants detected were in genes that have been previously proven to cause POI. One was homozygous variant in *FIGLA* gene and the other was homozygous variant in *PSMC3IP* gene. Heterozygous variants were detected in *PROK2, WDR11* and *CHD7* associated with hypogonadotropic hypogonadism, but these variants are insufficient to contribute to the POI phenotype.

**Conclusion:**

Genetic panels based on next generation sequencing analysis technologies can be used to determine the molecular genetic diagnosis of POI, which has a highly heterogeneous genetic basis.

## INTRODUCTION

Primary ovarian insufficiency (POI) is a general term used to describe depletion or dysfunction of ovarian follicles with cessation of menses before age 40. Although no accurate epidemiological data exists, approximately 1% of women are affected by the age of 40 and the frequency increases with age (1,2). POI can be isolated (non-syndromic), or as one component of a pleiotropic genetic syndrome. The disorder is highly heterogeneous in etiology. It can result from a small pool of primordial follicles, disturbances of follicle function, and premature follicle depletion due to accelerated atresia. A wide spectrum of causes has been considered, including genetic, autoimmune, infectious, or iatrogenic. Irrespectively, the majority remains to be elucidated (3).

Genetic causes of POI account for approximately 20%-25% of patients. Chromosomal abnormalities have long been considered as a component of POI etiology, and could explain 10%-15% of POI cases (4). Yet identifying precise causative genes for POI has been challenging. Over the past few decades, numerous candidate genes emerged but few were incorporated as causative with functional validation. Very recent approaches using next generation sequencing (NGS) in large POI pedigrees, have led to new causatives being identified and candidates being proposed. While the lists are expanding, the knowledge of POI genetics remains insufficient. In addition, the fact that no single underlying dominant gene deficiency could explain the disorder further supports the genetic heterogeneity of POI etiology.

In this study, we applied next generation sequencing (NGS) in twenty-three individuals with POI for whom clinical phenotyping and molecular studies had not previously yielded a molecular diagnosis. We tried to explain the clinical phenotype in cases by identifying variants in known genes and attempting to identify candidate genes associated with POI.

## SUBJECTS AND METHODS

### Patients

Twenty-three Turkish cases with primary ovarian insufficiency who were followed up at the Pediatric Endocrinology, Pediatric Genetics, and Medical Genetics Departments at Ege University Medical Faculty were evaluated, and all patients had previously a standard metaphase karyotype (450-550 bands) of 46, XX. Individuals with primary or secondary amenorrhea before the age of 40, with elevated FSH (>40 IU/mL) and low anti-Müllerian hormone levels (<0.03 ng/mL) were enrolled into the study. The patients with anti-cancer therapy, pelvic surgery, ovarian infection and/or autoimmune disease history were excluded. A pedigree was drawn for each patient showing at least 3 generations. Patients’ demographic data and laboratory and radiologic evaluations were obtained from file records.

### Next generation sequencing

TruSight^TM^ Exome focuses on a subset of disease-causing genes shown to be important in specific inherited conditions. The coding region of more than 4,800 genes, including a total of sixty-four non-syndromic POI genes (*BMP15, AR*, *FOXO4, POF1B*, *DACH2, PGRMC1*, *FMR1, INHA*, *NOBOX, SR5A1*, *MCM8, MCM9*, *AMHR2, AMH*, *KITLG, MSH4*, *LHX8, TGFBR3*, *GPR3, WNT4*, *FIGLA, FSHR*, *INHBB, FOXL2*, *PTEN, NANOS1*, *NANOS2, NANOS3*, *KIT, SKP2*, *PRLR, FST*, *GDF9, POUFF1*, *MSH5, FOXO1*, *CITED2, INHBA*, *SOHLH1, SOHLH2*, *SAL4, NOG*, *CYP19A1, LHCGR*, *CYP17A1, BMPR1B*, *BRCA1, IGSF10*, *MND1, MRPS22*, *PAD16, NUP107*, *PSMC3IP, PNPLA7*, *PRKD1, PDE8A*, *COPD7, ATAD3A*, *DDR2, NR2C2*, *PCYT1B, SRC*, *THBS1, SH2B1*), are covered by the TruSight^TM^ Exome panel (5). The genes that are known or suspected to be involved in hypergonadotropic hypogonadism were selected (6). The Illumina MiSeq platform was used to perform NGS. Sequencing data were analyzed using Illumina VariantStudio variant analysis software and Integrative Genomics Viewer (7).

### Data analysis

All genes in this panel were annotated. The homozygous or compound heterozygous variants of POI-causing genes with a frequency of less than 0.5% in public databases (e.g., NCBI dbSNP build141 [http://www.ncbi.nlm.nih.gov/SNP/], 1000 Genomes Project [http://www.1000genomes.org/], Exome Aggregation Consortium [http://exac.broadinstitute.org/], and NHLBI Exome Sequencing Project Exome Variant Server [http://evs.gs.washington.edu/EVS/]) were selected. The impact of the variants on the protein structure was identified using several in silico prediction tools, such as MutationTaster (8), Polyphen-2 (9), and SIFT (10). Conservation of residues across species was evaluated using PhyloP algorithm (10), and identified genomic evolutionary rate profiling variants were then categorized according to the American College of Medical Genetics recommendations (11). All variants found in the study (single nucleotide variations and insertions or deletions) were evaluated with the dbSNP database, and the average numbers and percentages of the variants reported in the dbSNP database were calculated.

### Confirmation and segregation analysis

The most likely disease-causing variants were confirmed using direct Sanger sequencing with ABI PRISM 3730 DNA Analyzer (Applied Biosystems) and BigDye Terminator Cycle Sequencing v3.1 Ready Reaction Kit (Life Technologies). Segregation analysis was also performed in the parents.

### Statement of ethics

Written informed consent form was provided by all participants or their parents. This study was approved by the Ege University Ethics Committee (Number: 19-3T/7, Date: 06.03.2019).

## RESULTS

Median age of the patients was 17.8 (min-max: 14.0-24.3) years, and 12 (52%) of the individuals admitted before the age of 18 years. Fourteen (60.8%) of the individuals had secondary amenorrhea. Fifteen (65%) of the cases had consanguineous parents. Two (8.6%) of the patients had family members with the same clinical features. Mean weight, height and BMI SDS were −0.5 ± 1.3SD, −0.3 ± 1.7SD, −0.0 ± 1.4 SD respectively. Four of the individuals had short stature and 3 of these patients received growth hormone treatment. Mean FSH level of the cases was 82.73 ± 35.21 IU/mL and anti-Müllerian hormone (AMH) levels of all patients were <0.03 ng/mL.

### NGS data

On average, 23 probands had 98.70, 93.33, and 87.17% of mappable bases represented by a coverage of at least 1, 10, and 20 reads, respectively. Considering only the sixty four POI genes included in TruSight TM Exome, the percentage of mappable bases represented by a coverage of at least 10, and 20 reads were 99.60, and 98.90%, respectively. An average read depth of 138.47 reads was achieved for 64 targeted genes. The average numbers of single-nucleotide variations and insertions or deletions in whole genes covered by the panel were 3,497 and 64.54, respectively. 93.99% of single-nucleotide variations and 60.01% of insertions or deletions were found in the db-SNP database.

### Variants in known POI genes

A total of 5 (21%) variants in 5 different genes (*FIGLA, PSMC3IP*, *PROK2, WDR11*, *CHD7*) were identified in 23 patients. Four variants were novel. All variants were predicted to be pathogenic by in silico analysis. Table 1 shows the variants found in NGS analysis.

**Table 1:** Molecular characteristics of the identified variants in the study group

Study Group	Country of origin	Consanguinity	Gene (transcript)	Variant	Zygosity	ACMG Interpretation	Frequency	dbSNP	SIFT	PolyPhen2	Mutation Taster	Reported
**Case 8**	Turkey	1st cousins	PSMC3IP(NM_016556.4)	c.215T>C (p.Phe72Ser) chr17- 40729241	Homozygous	VUS (PM2+PP3)	0.000004	rs1363637383	Benign	Possibly Damaging (0.767)	Disease Causing	Novel
**Case 14**	Turkey	1st cousins	FIGLA(NM_001004311.3)	c.339T>G (p.Tyr113Ter) chr2- 71014826	Homozygous	LP (PVS1+PMS2)	0	NA	NA	NA	Disease Causing	Novel
**Case 2**	Turkey	No	WDR11(NM_018117.12)	c.449C>T (p.Thr150Ile) chr10- 122619717	Heterozygous	VUS (PM2)	0.000004	rs577805675	Uncertain (0.002)	Uncertain (0.54)	Disease Causing	Novel
**Case 6**	Turkey	No	PROK2(NM_001126128.2)	c.217C>T (p.Arg73Cys) chr3- 71830623	Heterozygous	LP (PM2+PP3+PP5)	0.000050	rs121434272	Uncertain (0.003)	Probably Damaging (1)	Disease Causing	12
**Case 20**	Turkey	No	CHD7(NM_017780.4)	c.5420A>G (p.Asn1807Ser) chr8- 61763067	Heterozygous	VUS (PM2+ PM1+ PP2)	0	NA	Uncertain (0.079)	Benign (0.057)	Disease Causing	Novel

Abbreviations: LP: likely pathogenic; PM: pathogenic moderate; PP: pathogenic supporting; PS: pathogenic strong; PVS: pathogenic very strong; VUS: variant of uncertain significance.

### PSMC3IP variant (Case 8)

A 19-year-old patient presented with the complaint of not having menstruation despite the development of secondary sexual characteristics. The patient's parents were first-degree cousins. Her parents were healthy; no other family members had similar clinical fetaures. On physical examination, her puberty stage was prepubertal. She had no other medical problems and lacked dysmorphic features. Her weight, height and BMI score were 65.2 kg, 168.8 cm, 22.8 kg/m^2^ respectively. Her initial FSH concentration was 112 IU/L. Uterus 26 × 8 × 15 mm, endometrium 2.6 mm, ovaries were not observed in pelvic ultrasonography and pelvic MRI was reported as uterus was anteverted 28 mm and hypoplasic, ovaries were hypoplasic. Homozygous c.215T>C (p.Phe72Ser) variant was found in the *PSMC3IP* gene by NGS analysis. This variant has not been reported previously. This gene is associated with AR Ovarian dysgenesis 3 (OMIM 614324) in OMIM.

### FIGLA variant (Case 14)

The 21 year-old individual admitted due to lack of puberty findings at the age of 17 years. She was born as a result of first-degree cousin marriage. Her 16-year-old sister had similar clinical phenotype. The patient's three uncles had children (azoospermia/oligospermia?) with treatment. On physical examination, she had axillary and pubic hair and prepubertal breast with lipomastia. She had no other medical problems and lacked dysmorphic features. Her weight, height and BMI score were 58 kg (0.17 sds), 163 cm (0.07 sds), and 21.8 kg/m^2^ (0.2 sds), respectively. Her initial FSH concentration was 70.3 IU/L. Ovaries and uterus were not visualized in pelvic ultrasonography and pelvic MRI was reported as uterus and ovaries were not seen (agenesis?) and there is an appearance that may belong to a rudimented vagina. Homozygous c.339T>G (p.Tyr113Ter) variant was found in the *FIGLA* gene by NGS analysis. This variant has not been reported previously. This gene is associated with AD Premature ovarian failure 6 (OMIM 612310) in OMIM.

### CHD7 (Case 20), PROK2 (Case 6), and WDR11 variants (Case 2)

The 23 year-old case admitted due to primary amenorrhea at 21 years of age. There was no consanguinity between her parents. She had no family members had similar clinical phenotype. On physical examination, her puberty stage was tanner stage 2 with no axillary and pubic hair. She had no other medical problems and lacked dysmorphic features. Her weight, height and BMI score were 98 kg, 178.8 cm, and 31.5 kg/m^2^, respectively. Her initial FSH concentration was 44.9 IU/L. Ovaries and uterus were not visualized in pelvic ultrasonography and pelvic MRI was reported as uterus was atrophic according to her age and ovaries were not seen. Heterozygous c.5420A>G (p.Asn1807Ser) variant was found in the *CHD7* gene by NGS analysis. This variant has not been reported previously. This gene is associated with AD Hypogonadotropic hypogonadism 5 with or without anosmia (OMIM 612370) in OMIM.

The 17-year-old patient presented with the complaint of not having menstruation despite the development of secondary sexual characteristics. The patient's parents were first-degree cousins. Her parents were healthy; no other family members had similar clinical fetaures. On physical examination, her puberty stage was tanner stage 5. Her weight, height and BMI score were 60.7 kg (0.74 sds), 158.3 cm (-0.61), and 24.22 kg/m^2^ (1.15 sds), respectively. Her initial FSH concentration was 114.9 IU/L. In pelvic ultrasonography, uterus 37 × 26 × 44 mm, endometrium 9 mm and the dimensions of the right and left ovaries were 17 × 12 mm and 17 × 12.5 mm were observed respectively. Heterozygous c.217C>T;p.Arg73Cys variant was found in *PROK2* gene by NGS analysis. This variant was reported previously (12). This gene is associated with AD Hypogonadotropic hypogonadism 4 with or without anosmia (OMIM 610628) in OMIM.

The 24 year-old individual admitted due to lack of puberty findings. She was born as a result of third-degree cousin marriage. Her 18 and 20-year-old sisters had similar clinical phenotype. On physical examination, she had axillary and pubic hair and prepubertal breast with lipomastia. She had no other medical problems and lacked dysmorphic features. Her weight, height and BMI score were 45.4 kg, 163.2 cm, and 17.05 kg/m^2^, respectively. Her initial FSH concentration was 63 IU/L. In pelvic ultrasonography, uterus 24 × 5 × 11 mm, and the dimensions of the right and left ovaries were 13 × 15 mm and 12 × 4 mm were observed respectively. Heterozygous c.449C>T; p.Thr150Ile was found in *WDR11* gene by NGS analysis. This variant has not been reported previously. This gene is associated with AD Hypogonadotropic hypogonadism 14 with or without anosmia (OMIM 614858) in OMIM.

## DISCUSSION

Most cases of isolated POI still appear sporadically, but the incidence of familial cases is 10% to 15%, indicating a significant genetic etiology (3). In this study, using the NGS panel, which included 64 known POI genes, pathogenic variants in five (21%) cases were detected. Variants, which were consistent with the clinical features of the patients, were found in 2 (8.6%) cases.

*FIGLA* is the first germ cell-specific transcription factor that has been shown to affect primordial follicle formation (13). *FIGLA* plays a crucial dual role in activation of oocyte-associated genes and in repression of spermassociated genes during normal postnatal oogenesis. During postnatal ovarian development, *FIGLA* activates the female phenotype and represses the male germ cell genetic hierarchies in growing oocytes (14). Recently, 18- and 14-year-old Chinese sisters presented with primary amenorrhea were reported. Whole-genome sequencing analysis identified a homozygous variant in the *FIGLA* gene. Their parents were first-degree cousins. The little sister had short stature. Their 40-year-old mother had regular mensturation period (15). Similary, our case with *FIGLA* variant was born as a result of first-degree cousin marriage and her sister had similar clinical features. The patient's uncles were likely to have azoospermia.

*PSMC3IP* is critical for homologous pairing, and homologous recombination in meiosis (6). In a consanguineous Palestinian family with five members affected by XX-GD, a shared homozygous variant in *PSMC3IP* was identified. The deletion variant abolished *PSMC3IP* activation of estrogen-driven transcription. Impaired estrogenic signaling can lead to ovarian dysgenesis both by affecting the follicular pool and by failing to counteract follicular atresia (16). Recently, another homozygous variant in *PSMC3IP* was reported in a consanguineous Yemeni family of four sisters with ovarian dysgenesis and a brother with azoospermia (17). These results revealed a critical role of *PSMC3IP* in both male and female germ cell development. There was consanguinity between the parents of our case with *PSMC3IP* variant, but there was no individual with similar clinical phenotype in the family.

Multilocus variation has increasingly explained blended phenotypic outcomes in disease cohorts (18,19). The clinical variability of POI has contributed to the hypothesis that ovarian development is also a clinical phenotype in which multilocus variation may explain apparent phenotypic variability (18). Disease-causing rare variants that have been rapidly brought to homozygosity instead of cleared by selection due to consanguineous marriage (18). Just as this could occur for a single disease locus, homozygosity of rare variants at more than one locus can occur (18,19). A case with POI clinical phenotype with CHD7 pathogenic variant was detected recently in a multicenter study (18). Involved mutational burden and dual molecular diagnosis of these genes (*CHD7, PROK2*, and *WDR11*) may have caused POI findings. However, the current study did not reveal variants that could contribute to oligogenic inheritance that could explain the genotype-phenotype correlation in patients with POI. As a result of further genetic analysis, such as whole-exome sequencing, possible additional autosomal recessive variants to existing variations will help to explain the clinical findings of POI in these patients.

For the patients in whom no causative variant in a gene in this panel was found, there are 2 other possibilities. First, deep intronic variants or large exonic deletions might be responsible for the underlying molecular etiology. Second, a variant in a gene that is not covered by our panel may be responsible. In this study, further investigations, such as copy number variation analysis of the data, multiplex ligation-dependent probe amplification, or whole-exome sequencing, are planned to perform in the patients with no variants.

In conclusion, ovarian development is complex, as is the maintenance of its physiological functions during germ cell production. The presence of direct and indirect effectors of ovarian development function shows that a single pathway does not sufficiently explain ovarian physiology. However, NGS of POI cohorts can elucidate molecular candidates involved in female gonadal development and decades of physiological function required for germ cell production. Identifying variants causative of POI provides patients with a molecular diagnosis and accurate counseling regarding recurrence risk. Furthermore, this molecular diagnosis can guide expectant management. The continual elucidation of disease genes in POI allows for a deeper understanding of the molecular physiology of ovarian development and function.
